# Olive pomace oil can improve blood lipid profile: a randomized, blind, crossover, controlled clinical trial in healthy and at-risk volunteers

**DOI:** 10.1007/s00394-022-03001-y

**Published:** 2022-09-24

**Authors:** Susana González-Rámila, Beatriz Sarriá, Miguel A. Seguido, Joaquín García-Cordero, Raquel Mateos, Laura Bravo

**Affiliations:** Institute of Food Science, Technology and Nutrition (ICTAN-CSIC), Spanish National Research Council (CSIC), José Antonio Nováis 10, 28040 Madrid, Spain

**Keywords:** Olive pomace oil, Oleic acid-rich sunflower oil, Cardiovascular health, Lipid profile, Endothelial function

## Abstract

**Purpose:**

This study aimed to assess the effect of dietary consumption of olive pomace oil (OPO) on blood lipids (primary outcome) and other cardiovascular disease (CVD) risk factors (blood pressure, inflammation and endothelial function as secondary outcomes).

**Methods:**

A randomized, controlled, blind, crossover intervention was carried out in healthy and at-risk (hypercholesterolemic) subjects. Participants consumed daily 45 g of OPO or high-oleic acid sunflower oil (HOSO) as control oil during 4 weeks.

**Results:**

OPO significantly reduced low-density lipoprotein cholesterol (LDL-C; *P* = 0.003) and apolipoprotein B (Apo B; *P* = 0.022) serum concentrations, and LDL/HDL ratio (*P* = 0.027) in healthy and at-risk volunteers. These effects were not observed with HOSO. Blood pressure, peripheral artery tonometry (PAT), endothelial function and inflammation biomarkers were not affected.

**Conclusions:**

Regular consumption of OPO in the diet could have hypolipidemic actions in subjects at cardiovascular risk as well as in healthy consumers, contributing to CVD prevention.

**Clinical trial registry:**

NCT04997122, August 8, 2021, retrospectively registered.

**Supplementary Information:**

The online version contains supplementary material available at 10.1007/s00394-022-03001-y.

## Introduction

Morbidity and mortality from cardiovascular diseases (CVD) are increasing and represent a major public health problem worldwide [1]. CVD are caused by multiple factors, variable such as arterial hypertension, dyslipidemia, type 2 diabetes mellitus (T2D), smoking or physical inactivity, with genetics, sex and age being non-modifiable factors also playing a role in the pathophysiology of CVD. It is known that a healthy diet and physical exercise can modulate the risk of CVD [1, 2]. In this regard, the Mediterranean Diet (MD) is considered a model of healthy eating, associated with longer life expectancy and with protective effects against chronic diseases [3].

Olive oil is an essential component of the MD, being its primary fat source [3]. Depending on the processing of the olives, different categories of olive oil are obtained: extra virgin (EVOO), virgin (VOO), refined (ROO) olive oils, and olive pomace oil (OPO), all having a high content of monounsaturated fatty acids (MUFA), mainly oleic acid. The phenolic content of EVOO and VOO make these oils one of the best sources of dietary fat, with protective effects against CVD, inflammation and cancer [3, 4]. However, the concentration of phenolic compounds is lower in ROO and OPO due to the refining process applied to both oils. OPO is obtained from the waste product remaining after mechanical extraction of VOO, composed of the olive skin, pulp and stone [5]. Despite its lower content in phenolic compounds, OPO is rich in other bioactive compounds like squalene, pentacyclic triterpenes (oleanolic (OA) and maslinic (MA) acids, erythrodiol and uvaol), tocopherols, sterols and aliphatic fatty alcohols [6]. Numerous in vitro and preclinical studies have been conducted with one or more of these so-called minor components of OPO, offering promising results on different cardiometabolic traits [7–9]. However, research on the health effects of OPO in humans is limited to the assessment of some pure minor components administered as drugs or food supplements [10–12] or as functional VOO enriched in some triterpenes [13, 14], yet to this date the effect of consuming OPO in the diet has not been studied.

To this end, an intervention study has been carried out to assess the possible beneficial role of consuming OPO as the main source of fat in the diet on serum lipid concentrations (primary outcome) and other biomarkers of cardiovascular health such as blood pressure, endothelial function and inflammation (secondary outcomes) in at-risk (hypercholesterolemic, HC) subjects. To further explore the potential effects of OPO, healthy normocholesterolemic (NC) volunteers were also included in the study.

## Materials and methods

### Study design and outcomes

The study was a randomized, blind, crossover, controlled clinical trial in free-living subjects. It consisted of two 4-week interventions with OPO and high-oleic acid sunflower oil (HOSO), preceded each by 3-week run-in or wash-out periods. The study timeline is shown in Supplementary Fig. 1S. HOSO was used as control oil because it has a similar MUFA content than OPO but with a different profile of minor components. During run-in and wash out, volunteers consumed sunflower oil (SO) and followed the same food restrictions indicated below.

The primary study outcomes were changes in fasting serum concentrations of total (TC) or low-density lipoprotein (LDL-C) cholesterol or triglycerides (TG) in at-risk HC subjects. Secondary outcomes included changes in biomarkers of endothelial function, such as concentrations of E- and P-selectin, intercellular (ICAM-1) and vascular (VCAM-1) cell adhesion molecules, endothelial nitric oxide synthase (eNOS) and, in the HC subjects, changes in peripheral artery tonometry (PAT). Other secondary outcomes were changes in systolic (SBP) and diastolic (DBP) blood pressure and in inflammatory biomarkers.

### Participants

Participants were men and women aged 18–55 years with a body mass index (BMI) between 18 and 25 kg/m^2^ selected on the basis of fasting serum TC and LDL-C concentrations. Specific inclusion criteria for at-risk volunteers were TC concentrations between 200 and 300 mg/dL and LDL-C between 135 and 175 mg/dL. Healthy volunteers had TC concentrations below 200 mg/dL and LDL-C below 135 mg/dL. Exclusion criteria included suffering from acute or chronic pathologies, except hypercholesterolemia for the risk group, having digestive disorders/pathologies (gastric ulcer, Crohn’s disease, etc.), smoking, pregnant women, vegetarians, on antibiotic treatment three months before starting the study or taking medication, hormones, vitamins or dietary supplements.

**Fig. 1:**
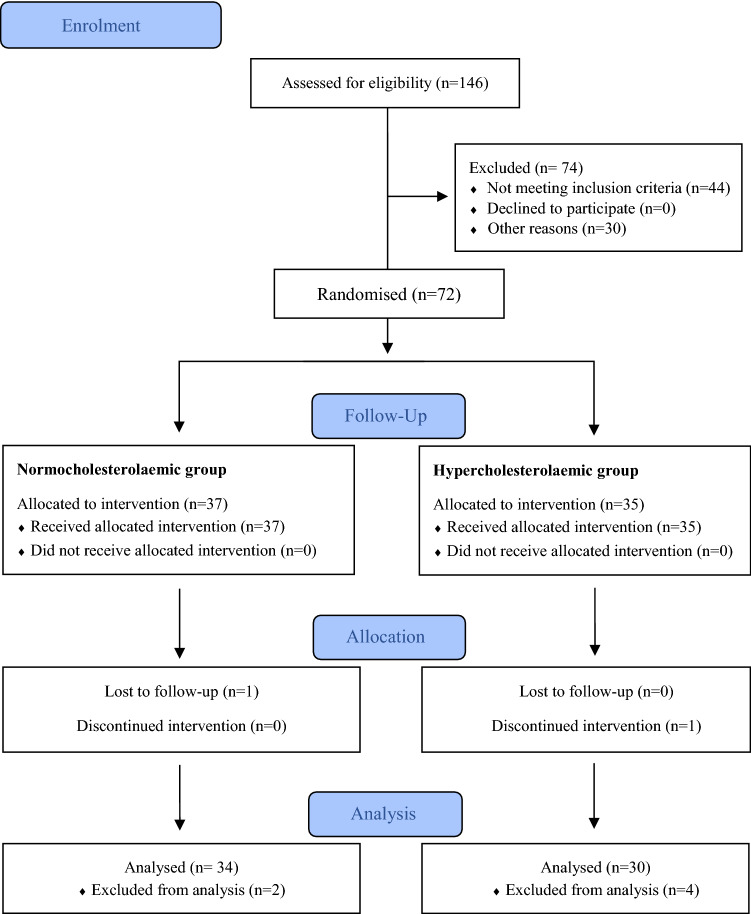
Study flowchart according to CONSORT (Consolidated Standards of Reporting Trials)

Subjects were recruited between November 2017 and January 2018 at the Institute of Food Science, Technology and Nutrition (ICTAN). Recruitment was carried out mainly at the campus of Complutense University of Madrid (UCM) by placing flyers in the faculties, research institutes, and through talks after lectures, also through websites and internet. Potential volunteers were informed by phone, e-mail or in person at ICTAN. Subjects who met the inclusion criteria were invited to an on-site visit and interviewed about their medical condition and dietary habits. They also provided a recent blood analysis to confirm TC concentrations or referred to a collaborating laboratory (Unilabs S.L., Madrid) for this analysis. This biomarker (TC concentration) was the main variable for allocating participants in the normocholesterolemic or hypercholesterolemic groups. Thus, of the 146 subjects interviewed, 72 volunteers were recruited, corresponding to 37 healthy subjects (NC group) and 35 at-risk subjects (HC group) (Fig. 1).

Randomization of participants was done by generating random numbers using Microsoft® Excel 2016 program. It was controlled by the health status (i.e. NC or HC) and within each group, subjects were allocated (to the OPO or HOSO interventions) to sequence order in a 1:1 ratio, due to the crossover design of the study. Assignment of codes to participants, randomization and allocation to each oil were carried out by different team members. Samples were coded according to the study visit number to conceal treatment allocation during handling and analysis. Oils (OPO, HOSO and SO) were bottled in un-marked bottles with different cap colours. Participants were blinded to the type of oil they consumed in each intervention phase. They were habitual consumers of VOO or EVOO and were not familiar with OPO and HOSO. Therefore, it can be assumed they were not able to infer which oil was used in each intervention stage despite differences in colour and taste of OPO and HOSO.

To estimate sample size, the G*Power 3.1.9.7 program was used. We considered TC concentration as the main variable and that the study design (randomized, blind, crossover, controlled intervention), in which the same subjects consumed the test (OPO) and control (HOSO) oils. Other premises considered were differences by treatment, a statistical power of 80%, a level of significance of 0.05, two tail, standard deviation of 25, mean of pre-post differences of 15 units, and an effect size of 0.54. Considering all these parameters, a final sample size of 30 volunteers was established for group. Finally, the number of participants were NC (normocholesterolemic) = 34 and HC (hypercholesterolemic) = 30.

### Interventions

The intervention was conducted during April–July 2018 at the Human Nutrition Unit (HNU) of ICTAN. Participants consumed 45 g/d of the corresponding oil (OPO or HOSO, and SO during run-in and wash-out, equivalent to 4–5 tablespoons). This amount of oil was calculated considering the nutritional objective established by the Spanish Society for Community Nutrition for the adult population, that recommends that MUFA intake covers 20% of the daily energy intake (equivalent to 44–67 g/d for 2000–3000 kcal/d, respectively) and assuming that no other oils were consumed during the study and that the intake of MUFA-rich foods was restricted (see below). Volunteers were asked to maintain their dietary and lifestyle habits unchanged during the study, replacing their usual cooking oil by the one provided in each phase of the study. They could use the oil for cooking, baking, frying, or raw as salad dressing or on toasts. One litre of the oil corresponding to each phase (OPO, HOSO or SO) was provided to each volunteer per week for family use, thus ensuring no other source of fat was used. Consumption of foods rich in mono- and polyunsaturated fat (olives, sunflower seeds, nuts, avocado, margarine, butter and mayonnaise, except if prepared with the study oils) was restricted.

During the study, participants attended the HNU five times. On week 1, before run-in, baseline characteristics of volunteers were recorded, measuring blood pressure and basic anthropometric parameters (height, weight and waist circumference). A fasting blood sample was obtained to measure basal biochemical and haematological parameters. Volunteers handed-in a 72 h detailed food intake record to obtain information on their basal dietary habits. Subsequent visits corresponded to the initial and final time of each 4-week intervention (Fig. 1S), when fasting blood samples were collected, blood pressure was measured, and body weight was controlled. Only in the HC group, PAT was measured.

### Dietary control and compliance

In the last week of each intervention and run-in/wash-out stages, volunteers were asked to complete a 24 h dietary questionnaire corresponding to the day before each control visit, and previously a 72 h detailed food intake record. Total calories, macronutrients, micronutrients and dietary fibre (DF) intake were evaluated using the program DIAL (Faculty of Pharmacy, UCM, Madrid, Spain).

Compliance with the food restrictions and correct intake of oils was controlled by weekly calling or emailing participants. Volunteers were provided with an incident and observation sheet where they could note down any anomalies or irregular developments.

### Blood collection and biochemical analysis

After an overnight fast, 25 mL of blood was collected in BD Vaccuette® tubes (Greiner Bio-One GmbH, Kremsmünster, Austria) with EDTA or without anticoagulant to obtain plasma and serum, respectively, that were separated by centrifugation and stored at − 80 °C until analysis.

Biochemical analysis in serum was performed following reference procedures recommended by the Spanish Society of Clinical Biochemistry and Molecular Pathology. Lipid metabolism biomarkers were determined using a Roche Cobas Integra 400 plus analyser (Roche Diagnostics, Mannheim, Germany). TC, TG and high-density lipoprotein cholesterol (HDL-C) were determined, along with apolipoproteins A1 (Apo A1) and B (Apo B). LDL and VLDL (very low-density lipoprotein) were calculated according to Friedewald formula, and Apo B/Apo A1, LDL/HDL and TC/HDL ratios were calculated [15]. Alanine aminotransferase (ALAT) and aspartate aminotransferase (ASAT) were analysed according to standard spectrophotometric procedures [16].

### Blood pressure

SBP and DBP were determined in triplicate with an OMRON® M2 HEM-7121-E sphygmomanometer (OMRON HEALTHCARE Co. Ltd., Kyoto, Japan) after resting in a sitting position for a minimum of 15 min and waiting 5 min between measurements.

### Endothelial function

Plasma concentrations of E-selectin (SEA029Hu), P-selectin (SEA569Hu) and eNOS (SEA868Hu) were determined by ELISA (Cloud-Clone Kit Corp., Katy, TX, USA) using a BioTek® Synergy™ HT Multi-Detection Microplate Reader controlled by BioTek®Gen5 software version 2.01.14 (BioTek Instruments, Winooski, VT, USA). Circulating concentrations of ICAM-1 and VCAM-1 were determined in serum with Bio-Plex® Pro Human Cytokine ICAM-1 and VCAM-1 kit (Bio-Rad, Hercules, CA, USA) using a MAGPIX™ Multiplex fluorescence reader operating with the Bio-Plex Pro Wash Station and the Bio-Plex Manager™ MP software for data processing (Luminex Corporation, Austin, TX, USA).

Considering that PAT is usually reduced in individuals with cardiovascular risk factors, the hyperemic response was assessed only in the HC subjects by measuring pulsatile arterial volume changes by finger plethysmography using an Endo-PAT 2000® equipment (Itamar Medical, Caesarea, Israel). Volunteers rested in a supine position for at least 10 min before starting measurements of finger pulse wave amplitude (PWA) pre- and post-occlusion of the blood flow in the study arm. The reactive hyperaemia index (RHI) was calculated as the ratio of PWA during reactive hyperaemia *vs* baseline [17]. RHI values were transformed into natural logarithms (lnRHI). Values below 0.51 and above 0.51 were defined as abnormal and normal, respectively.

### Inflammatory biomarker

Serum samples were used to determine circulating concentrations of the cytokines interleukin (IL)-1β, IL-2, IL-4, IL-5, IL-6, IL-7, IL-8, IL-10, IL-12, IL-13, IL-17A, interferon-gamma (IFNγ), and tumour necrosis factor alpha (TNFα), monocyte chemoattractant protein 1 (MCP-1), macrophage inflammatory protein 1 beta (MIP-1β), granulocyte colony-stimulating factor (G-CSF), and granulocyte monocyte colony-stimulating factor (GM-CSF) in serum samples using the Bio-Plex® Pro Human Cytokine 17-plex Assay kit (Bio-Rad, Hercules, CA, USA) in the MAGPIX™ Multiplex fluorescence reader and Bio-Plex Manager™ MP software (Luminex Corporation, Austin, TX, USA). High-sensitive C-reactive protein (CRP) was measured in serum with an automatized ultrasensible turbidimetric method (AU2700 Chemistry Analyzer, Olympus Corp., Japan).

### Chemical characterization of the study oils

The oils used in the study were provided by Interprofesional del Aceite de Orujo de Oliva (ORIVA) and analysed according to standardized methods: ISO 12228–2:2014 method for the determination of sterols; Regulation (EEC) No. 2568/91 Annex V for triterpenic alcohols; Regulation (EEC) No. 2568/91 Annex XIX for aliphatic alcohols; Regulation (EEC) No. 2568/91 Annex X for fatty acids; and ISO 9936:2016 for tocopherols and tocotrienols. Triterpenic acids were analysed following the method of Pérez-Camino & Cert [18], and squalene was determined by gas chromatography [19]. Phenols were analysed by high-performance liquid chromatography according to Mateos et al. [20]. Supplementary Table 1S shows the detailed composition of the oils. OPO and HOSO were rich in MUFA, with an oleic acid (C18:1n9) content of 71.0% and 76.5%, respectively. SO is predominantly a polyunsaturated fat, with a linoleic acid (C18:2n6) content of 58.6%, also presenting relevant amounts of C18:1n9 (29.6%). Tocopherols and sterols were higher in HOSO (583 mg/kg and 3040 mg/kg, respectively) and SO (562 mg/kg and 3315 mg/kg, respectively) than OPO (389 mg/kg tocopherols and 2839 mg/kg sterols). However, OPO showed a higher content of aliphatic alcohols (978 mg/kg), squalene (799 mg/kg), triterpenic acids (196.5 mg/kg), and triterpenic alcohols (886.6 mg/kg) compared to the low or no content of these minor components in HOSO (32 mg/kg aliphatic alcohols, 87 mg/kg squalene and < 2 mg/kg triterpenic acids and alcohols) and SO (26 mg/kg aliphatic alcohols, 117 mg/kg squalene and < 2 mg/kg triterpenic acids and alcohols) (Supplementary Table 1S).

### Statistical method

For the statistical design, the following factors were taken into account as two fixed effects: group (normocholesterolemic/hypercholesterolemic) and treatment (OPO/HOSO, repeated measures), and a random effect to consider the order of oil intake (starting with OPO or HOSO within each group).

#### Statistical models

The statistical models applied for analyzing the results of this study were:

1. General linear repeated measures model to study energy, macronutrient and micronutrient intake throughout the study, considering that the order of intake of the test and control oils did not matter since it would not affect the overall dietary pattern of the volunteers. In each group (normocholesterolemic and hypercholesterolemic), baseline, initial (pre-treatment) and final (post-treatment) results with OPO and HOSO were compared. Results are shown as mean ± standard deviation (SD).

2. A linear mixed model was applied to the relative changes from initial [(final value–initial value)/initial value] of each variable. This statistical model allows the data to present a correlated and non-constant variability to take into account the order of intake of the oils. The statistical model was full factorial, considering group (normocholesterolemic hypercholesterolemic), treatment oil (OPO and HOSO) and interaction group*treatment, order of intake was a random effect. Results are shown as relative changes from initial value ± standard deviation (SD) and are expressed as a percentage.

The initial (pre-treatment) mean values were included in the tables as a reference value for relative change from the initial. All measurements (except dietary control) were performed at least in duplicate unless otherwise stated. Before statistical analysis, data normality of distribution was verified by the Kolmogorov–Smirnov test and a box-plot analysis was performed for all variables. In addition, the Bonferroni test (within each group) was performed to compare pairwise the effect of the intake of each oil (OPO and HOSO). The significance level was set at *P* < 0.05. Data were analysed using SPSS software (version 27.0; SPSS, Inc., IBM Company).

## Results

### Participants’ characteristics, dietary control and compliance

Although variability due to sex was not an objective of the present study, we aimed at recruiting a similar number of men and women; thus, of the 72 volunteers recruited, 35 were men and 37 women, yet finally more women (34) than men (30) completed the study. One NC volunteer and one HC participant withdrew due to personal reasons. Thus, 36 NC and 34 HC subjects completed the study; however, 2 NC and 4 HC participants were excluded from data analysis due to abnormal values in their blood tests (e.g. very high aminotransferase, CRP or TG concentrations at some control tests). Thus, 64 volunteers completed the study and were analysed (Fig. 1). Due to technical reasons, E-selectin could only be analysed in samples from 17 NC and 16 HC patients. The baseline characteristics of the participants who completed the study are shown in Table 1. No adverse events were reported.

**Table 1:** Baseline characteristics of participants

	Normocholesterolemic *n* = 34	Hypercholesterolemic *n* = 30
Men, *n*	13	17
Women, *n*	21	13
Age, y	35 ± 70	44 ± 52
BMI, kg/m^2^	24 ± 17	24 ± 17
Waist circumference, cm	77 ± 12	83 ± 12
Total-cholesterol, mg/dL	177 ± 29	239 ± 29
LDL-cholesterol, mg/dL	100 ± 23	150 ± 35
Systolic blood pressure, mmHg	118 ± 12	123 ± 12
Diastolic blood pressure, mmHg	73 ± 12	78 ± 12

Values represent mean ± SD. *BMI* body mass index, *LDL* low-density lipoprotein

Table 2 shows energy, macronutrient, DF, and micronutrient intake. Macronutrient and energy intake remained constant, with no statistically significant differences due to the consumption of the different oils (*P* < 0.05). Mean energy intake throughout the dietary intervention ranged from 2019 to 2261 kcal/d, which was slightly below normal limits for healthy adults (2185–3000 kcal/d) according to the recommended intakes for the Spanish population [21]. Protein, carbohydrate and fat represented 17.4%, 38.7%, and 39.9%, respectively, of total caloric intake. According to the recommended daily intake and nutritional objectives for the Spanish population, macronutrients were not within reference ranges of 10–15% for proteins, 50–60% for carbohydrates and < 35% for lipids [21].

**Table 2:** Energy intake and dietary components during the intervention trial with olive pomace oil (OPO) and high oleic sunflower oil (HOSO)

	Normocholesterolemic *n* = 34					*P* value		Hypercholesterolemic *n* = 30				*P* value
		OPO		HOSO				OPO		HOSO		
	Baseline	Initial	Final	Initial	Final	Oil^2^	Baseline	Initial	Final	Initial	Final	Oil^2^
Energy (kcal/day)	2168 ± 584	2019 ± 484	2076 ± 571	2198 ± 484	2086 ± 92	0.235	2120 ± 340	2250 ± 520	2123 ± 482	2261 ± 520	2084 ± 851	0.158
Proteins (g/day)	93 ± 23	85 ± 23	91 ± 29	96 ± 23	91 ± 5	0.354	94 ± 22	95 ± 22	99 ± 27	98 ± 22	89 ± 27	0.291
Carbohydrates (g/day)	204 ± 58	198 ± 58	197 ± 70	209 ± 58	204 ± 10	0.842	207 ± 49	215 ± 71	207 ± 60	214 ± 66	212 ± 88	0.891
Lipids (g/day)	100 ± 35	90 ± 29	94 ± 35	100 ± 29	92 ± 5	0.228	91 ± 16	102 ± 27	90 ± 33	102 ± 33	88 ± 33	0.209
SFA (g/day)	33 ± 12^a^	26 ± 12^b^	29 ± 12^ab^	29 ± 12 ^ab^	27 ± 2 ^ab^	0.012	29 ± 5^a^	29 ± 11^b^	27 ± 11^ab^	30 ± 11^ab^	24 ± 11^ab^	0.036
MUFA (g/day)	45 ± 17^a^	28 ± 12^b^	45 ± 17^a^	31 ± 12^b^	46 ± 3^a^	< 0.001	41 ± 11^a^	32 ± 11^b^	43 ± 16^a^	32 ± 11^b^	46 ± 22^a^	< 0.001
PUFA (g/day)	14 ± 6^a^	28 ± 12^b^	13 ± 6^ac^	30 ± 12^b^	11 ± 1^b^	< 0.001	13 ± 5^a^	31 ± 11^b^	12 ± 5^a^	31 ± 16^b^	10 ± 5^a^	< 0.001
Cholesterol (mg/day)	328 ± 134	319 ± 152	331 ± 152	365 ± 152	335 ± 41	0.501	339 ± 153	330 ± 197	382 ± 208	334 ± 137	307 ± 159	0.131
Dietary fibre (g/day)	23 ± 12	22 ± 6	23 ± 12	23 ± 6	22 ± 1	0.940	25 ± 11	26 ± 11	25 ± 11	28 ± 11	23 ± 11	0.064
Vitamin E (mg/day)	9 ± 6^a^	24 ± 12^b^	20 ± 6^c^	26 ± 6^b^	17 ± 1^c^	< 0.001	10 ± 5^a^	28 ± 16^b^	21 ± 5^c^	27 ± 16^b^	19 ± 5^c^	< 0.001

Values represent mean ± SD. Data were analysed using a general linear repeated measures model. According to the Bonferroni test, superscripts correspond to significant differences within the NC or HC group. *P* values correspond to the effect of taking OPO or HOSO. Significance level was *P* < 0.05. *SFA* saturated fatty acid, *MUFA* monounsaturated fatty acid, *PUFA* polyunsaturated fatty acid

^2^Oil (baseline vs OPO vs HOSO)

Baseline data showed that volunteers’ habitual diet was rich in MUFA, with high intake of saturated fatty acids (SFA), between 24 and 33 g/d (equivalent to 11–13% of total energy, higher than the 7% recommended intake). SFA intake showed significant differences (*P* = 0.006) after the intervention with OPO and HOSO, decreasing in comparison with baseline values. As expected, MUFA intake decreased after consumption of SO during run-in/wash-out in both groups (*P* < 0.001), as shown by the MUFA values at the beginning of each intervention (between 28 and 32 g/d, equivalent to 11.8% and 13.5% of the total energy, respectively). However, values returned to those of the basal diet after the dietary intervention with OPO (45 g/d and 43 g/d, equivalent to 18.9% and 18.1% of the total energy in the NC and HC groups, respectively) and HOSO (46 g/d equivalent to 19.3% of total energy in the NC and HC groups) recovering recommended values of MUFA intake (> 17%). As for polyunsaturated fatty acids (PUFA), consumption of SO, rich in linoleic acid, increased the total intake of PUFA (*P* < 0.001) after run-in/wash-out (between 28 g/d and 31 g/d, equivalent to 11.8% and 13.0% of the total energy, respectively), returning to basal, normal intakes (3–6% of total energy) during OPO (13 g/d and 12 g/d, equivalent to 5.5% and 5.0% of the total energy in the NC and HC groups, respectively) and HOSO (11 g/d and 10.3 g/d, equivalent to 4.6% and 4.3% of the total energy in the NC and HC groups, respectively) interventions. There were no significant effects of OPO and HOSO on cholesterol and DF, with intakes close to recommended values (< 300 mg/d for cholesterol and 25–30 g/d for DF). Vitamin and mineral intake remained constant, except for vitamin E. In both groups of volunteers, basal intake of Vitamin E was below values recommended for the Spanish adult population, set at 13 and 11 mg/day for men and women, respectively [21]. The intake of vitamin E increased during the intervention, with values ranging between 17 and 28 mg/d, showing significant variations due to the effect of the oil (*p* < 0.001) (Table 2). This higher intake of vitamin E reflected the α-tocopherol content of the oils (OPO, HOSO and SO) (Table 1S). The pattern of lipids, fatty acid and vitamin E consumption suggests good compliance with the intervention and dietary recommendations.

### Lipid profile and liver function

The effect of dietary treatment on volunteers’ lipid profile is shown in Table 3. As can be seen, no statistically significant differences were observed in the interaction of oil with group for any of the parameters, including VLDL (*P* = 0.058), which was close to reaching the significance level.

**Table 3:** Effect of olive pomace oil (OPO) and high oleic sunflower oil (HOSO) consumption on lipid profile and liver function

	Normocholesterolemic *n* = 34		Hypercholesterolemic *n* = 30		*P* value		
	OPO	HOSO	OPO	HOSO	Oil	N/H	N/H*Oil
Total-cholesterol (mg/dL)							
Initial	172 ± 119	164 ± 125	225 ± 146	221 ± 158			
Relative changes from initial (%)	− 1.4 ± 7.4	3.4 ± 8.9	− 0.2 ± 6.9	2.2 ± 11.1	0.064	0.842	0.354
Triglycerides (mg/dL)							
Initial	79 ± 196	72 ± 148	90 ± 229	101 ± 319			
Relative changes from initial (%)	− 0.5 ± 23.4	10.4 ± 35.3	8.3 ± 22.7	4.5 ± 38.5	0.968	0.785	0.130
HDL-cholesterol (mg/dL)							
Initial	59 ± 73	58 ± 58	61 ± 84	61 ± 82			
Relative changes from initial (%)	1.9 ± 10.7	2.3 ± 10.6	4.1 ± 12.3	3.3 ± 11.7	0.859	0.409	0.632
LDL-cholesterol (mg/dL)							
Initial	97 ± 110	94 ± 113	146 ± 167	140 ± 183			
Relative changes from initial (%)	− 2.7 ± 12.9	4.2 ± 12.9	− 4.3 ± 11.1	4.2 ± 19.3	0.003	0.688	0.803
VLDL-cholesterol (mg/dL)							
Initial	18 ± 101	14 ± 30	18 ± 46	20 ± 63			
Relative changes from initial (%)	− 4.4 ± 26.9	5.5 ± 28.1	6.7 ± 32.0	− 3.0 ± 31.3	0.984	0.775	0.058
Apo A1 (mg/dL)							
Initial	153 ± 150	146 ± 102	155 ± 115	157 ± 129			
Relative changes from initial (%)	− 1.8 ± 7.8	1.9 ± 9.2	2.2 ± 9.6	1.9 ± 9.1	0.632	0.312	0.089
Apo B (mg/dL)							
Initial	78 ± 77	76 ± 84	112 ± 115	109 ± 120			
Relative changes from initial (%)	− 2.9 ± 9.6	0.7 ± 9.1	− 6.5 ± 8.6	− 0.5 ± 13.4	0.022	0.192	0.649
Apo B/Apo A1 (mg/dL)							
Initial	0.5 ± 0.7	0.5 ± 0.7	0.7 ± 1.1	0.7 ± 1.2			
Relative changes from initial (%)	− 1.2 ± 14.3	− 2.5 ± 15.3	− 8.5 ± 8.7	− 0.5 ± 13.4	0.318	0.210	0.130
LDL/HDL							
Initial	2 ± 3	2 ± 3	3 ± 6	2 ± 6			
Relative changes from initial (%)	− 3.7 ± 15.4	3.0 ± 16.3	− 6.2 ± 15.1	1.7 ± 20.2	0.027	0.473	0.837
TC/HDL							
Initial	3 ± 4	3 ± 4	4 ± 7	4 ± 6			
Relative changes from initial (%)	− 3.1 ± 11.9	1.8 ± 9.7	− 3.1 ± 11.2	0.5 ± 11.3	0.098	0.437	0.502
ALAT (UI/L)							
Initial	23 ± 10	21 ± 9	29 ± 13	27 ± 15			
Relative changes from initial (%)	116.8 ± 43.5	121.2 ± 67.7	122.3 ± 141	114.6 ± 36.6	0.739	0.466	0.739
ASAT (UI/L)							
Initial	24 ± 8	23 ± 6	26 ± 9	26 ± 8			
Relative changes from initial (%)	107.2 ± 28.3	110.1 ± 67.3	118.0 ± 71.2	106.0 ± 20.9	0.947	0.838	0.523

Values represent the initial (pre-treatment) mean values and relative changes from initial values expressed as percentage ± SD. Relative changes from initial values were calculated from initial and final values as [(final value-initial value)/initial value] and analysed using a linear mixed model. *P* values in the first column correspond to the effect of taking OPO or HOSO, those of the penultimate column to the effect of the group (NC or HC), and the last column to the interaction of oil and group. Significance level was *P* < 0.05. *Apo* apolipoprotein, *ALAT* alanine aminotransferase, *ASAT* aspartate aminotransferase

Intervention for 4 weeks with OPO showed significant changes (*P* = 0.003) on LDL-C, with a decrease of 2.7% in NC and 4.3% in HC. In turn, HOSO resulted in increased rates of around 4% LDL-C in both groups. Apo B also showed a significant decrease (*P* = 0.022) after OPO consumption in NC (− 2.9 ± 9.6%) and HC (-6.5 ± 8.6%) volunteers. LDL/HDL ratio also showed significant variations (*P* = 0.027) when initial (pre-treatment) and final (post-treatment) values were compared after OPO and HOSO intake. A moderate decrease was observed in TC after OPO consumption (− 1.4 ± 7.4% in NC and − 0.2 ± 6.9 in HC), whilst an increase was observed after HOSO intake in both groups (3.48 ± 1.53% in NC and 2.17 ± 2.03% in HC), although these changes did not reach the level of significance (*P* = 0.064) due to the great interindividual variability. Comparison on the response of these biomarkers in NC and HC volunteers after consuming OPO and HOSO is shown in Supplementary Fig. 2S. The rest of the biomarkers analysed were not affected.

### Blood pressure

As shown in Table 4, SBP and DBP showed no significant changes after dietary intervention with OPO and HOSO in both population groups studied. According to the American Heart Association, all volunteers maintained normal values of DBP and SBP (< 80 mmHg and < 120 mmHg, respectively).

**Table 4:** Effect of olive pomace oil (OPO) and high oleic sunflower oil (HOSO) consumption on blood pressure^1^

	Normocholesterolemic *n* = 34		Hypercholesterolemic *n* = 30		*P* value		
	OPO	HOSO	OPO	HOSO	Oil	N/H	N/H* Oil
Systolic BP (mmHg)							
Initial	113 ± 60	117 ± 57	116 ± 68	118 ± 61			
Relative changes from initial (%)	0.1 ± 4.6	− 0.2 ± 4.6	− 2.0 ± 6.8	− 0.4 ± 7.5	0.288	0.573	0.383
Diastolic BP (mmHg)							
Initial	74 ± 46	76 ± 46	78 ± 53	77 ± 55			
Relative changes from initial (%)	2.1 ± 8.6	0.8 ± 8.7	− 1.0 ± 9.0	3.3 ± 10.2	0.730	0.363	0.098

^1^ Values represent the initial (pre-treatment) mean values and relative changes from initial values expressed as percentage ± SD. Relative changes from initial values were calculated from initial and final values as [(final value-initial value)/initial value] and analysed using a linear mixed model. *P* values in the first column correspond to the effect of taking OPO or HOSO, those of the penultimate column to the effect of the group (NC or HC), and the last column to the interaction of oil and group. Significance level was *P* < 0.05. *BP* blood pressure

### Biomarkers of endothelial function

Endothelial molecules E-selectin, P-selectin, ICAM-1, and VCAM-1 did not show significant changes (*P* > 0.05) due to product effect (OPO and HOSO) (Table 5). However, an apparent tendency to decrease in E-selectin levels after OPO (− 2.6 ± 74.1% in NC; -9.0 ± 52.4% in HC) and HOSO (− 32.7 ± 66.3% in NC; − 2.0 ± 92.2% in HC) intake was observed (*P* = 0.347). Circulating eNOS increased in both groups, particularly after OPO intervention in healthy subjects (58.0 ± 150.8%), although these changes were not statistically significant. Finally, PAT determined in HC subjects did not differ in any treatment (*P* > 0.05).

**Table 5:** Effect of olive pomace oil (OPO) and high oleic sunflower oil (HOSO) consumption on biomarkers endothelial function

	Normocholesterolemic *n* = 34		Hypercholesterolemic *n* = 30		*P* value		
	OPO	HOSO	OPO	HOSO	Oil	N/H	N/H* Oil
eNOS (ng/mL)							
Initial	0.3 ± 2.3	0.5 ± 5.8	0.5 ± 5.5	0.6 ± 5.5			
Relative changes from initial (%)	58.0 ± 150.8	10.0 ± 138.2	38.7 ± 134.3	37.5 ± 92.3	0.827	0.493	0.070
E-selectin (ng/mL)							
Initial	6.3 ± 9.3	6.2 ± 7.6	7.3 ± 7.1	6.8 ± 6.6			
Relative changes from initial (%)	− 2.6 ± 74.1	− 32.7 ± 66.3	− 9.0 ± 52.4	− 2.0 ± 92.2	0.347	0.378	0.157
P-selectin (ng/mL)							
Initial	103 ± 59	95 ± 49	116 ± 57	107 ± 63			
Relative changes from initial (%)	18.3 ± 59.0	20.6 ± 65.3	11.4 ± 47.8	22.0 ± 61.4	0.863	0.588	0.993
ICAM-1 (ng/mL)							
Initial	1512 ± 365	1483 ± 379	1545 ± 603	1630 ± 564			
Relative changes from initial (%)	− 1.5 ± 25.9	2.1 ± 21.2	7.3 ± 56.6	− 1.2 ± 37.9	0.609	0.552	0.189
VCAM-1 (ng/mL)							
Initial	7098 ± 2917	6762 ± 3388	6299 ± 3139	6472 ± 3195			
Relative changes from initial (%)	3.6 ± 33.7	6.75 ± 40.0	15.7 ± 61.3	0.3 ± 54.1	0.480	0.753	0.277
PAT (LnRHI)^2^							
Initial	–	–	0.6 ± 1.4	0.5 ± 1.3			
Relative changes from initial (%)	–	–	29.5 ± 64.1	20.8 ± 49.9	0.484		–

Values represent the initial (pre-treatment) mean values and relative changes from initial values expressed as percentage ± SD. Relative changes from initial values were calculated from initial and final values as [(final value-initial value)/initial value] and analysed using a linear mixed model. *P* values in the first column correspond to the effect of taking OPO or HOSO, those of the penultimate column correspond to the effect of the group (NC or HC), and the last column to the interaction of oil and group. Significance level was set at *P* < 0.05. *eNOS* endothelial nitric oxide synthase, *E-selectin* endothelial selectin, *P-selectin* platelet selectin, *ICAM-1* intercellular adhesion molecule 1, *VCAM-1* vascular cell adhesion molecule 1

^2^ PAT: peripheral arterial tonometry

### Inflammatory biomarkers

According to the linear mixed model, inflammatory biomarkers did not show significant changes due to OPO and HOSO intervention (Table 6). However, when the relative changes from initial values were compared between NC and HC groups, IL-7 (*P* = 0.014) and IL-12 (*P* = 0.027) showed significant differences.

**Table 6:** Effect of olive pomace oil (OPO) and high oleic sunflower oil (HOSO) consumption on inflammatory biomarkers

	Normocholesterolemic *n* = 34		Hypercholesterolemic *n* = 30		*P* value		
	OPO	HOSO	OPO	HOSO	Oil	N/H	N/H* Oil
CRP (mg/dL)							
Initial	0.1 ± 0.3	0.1 ± 0.2	0.2 ± 0.4	0.1 ± 0.2			
Relative changes from initial (%)	128.8 ± 4.1	39.2 ± 1.7	136.9 ± 10.4	237.0 ± 6.0	0.999	0.127	0.380
IL-1β (pg/mL)							
Initial	0.7 ± 0.4	0.7 ± 0.3	0.9 ± 1.1	1.0 ± 0.6			
Relative changes from initial (%)	5.1 ± 50.1	9.9 ± 39.9	3.9 ± 31.3	− 10.0 ± 31.9	0.317	0.503	0.080
IL-2 (pg/mL)							
Initial	9 ± 6	9 ± 4	9 ± 5	11 ± 5			
Relative changes from initial (%)	− 0.9 ± 36.3	2.6 ± 26.3	24.0 ± 65.5	− 7.7 ± 51.0	0.175	0.806	0.043
IL-4 (pg/mL)							
Initial	7 ± 3	8 ± 2	10 ± 5	10 ± 5			
Relative changes from initial (%)	1.6 ± 27.1	2.6 ± 22.0	2.4 ± 27.1	10.1 ± 35.9	0.226	0.610	0.662
IL-6 (pg/mL)							
Initial	5 ± 4	5 ± 4	5 ± 4	6 ± 4			
Relative changes from initial (%)	− 6.5 ± 23.0	2.3 ± 36.8	13.9 ± 59.9	1.4 ± 35.6	0.901	0.131	0.162
IL-7 (pg/mL)							
Initial	52 ± 50	54 ± 47	50 ± 44	50 ± 50			
Relative changes from initial (%)	− 0.3 ± 27.1	− 3.6 ± 21.4	9.3 ± 30.2	11.6 ± 32.1	0.745	0.014	0.828
IL-8 (pg/mL)							
Initial	14 ± 8	15 ± 6	14 ± 10	16 ± 13			
Relative changes from initial (%)	18.7 ± 59.6	1.2 ± 33.6	18.3 ± 57.6	5.6 ± 42.5	0.133	0.792	0.839
IL-10 (pg/mL)							
Initial	15 ± 10	15 ± 9	16 ± 8	17 ± 9			
Relative changes from initial (%)	− 3.1 ± 34.2	3.4 ± 23.6	3.3 ± 37.9	− 0.9 ± 32.1	0.606	0.909	0.386
IL-12 (p70) (pg/mL)							
Initial	7 ± 2	6 ± 2	13 ± 28	12 ± 18			
Relative changes from initial (%)	12.9 ± 61.5	8.7 ± 57.4	− 1.4 ± 23.1	− 12.5 ± 50.3	0.937	0.027	0.378
IL-13 (pg/mL)							
Initial	4 ± 2	4 ± 1	5.5 ± 4	5.9 ± 4			
Relative changes from initial (%)	5.3 ± 37.8	12.3 ± 48.1	21.6 ± 65.1	22.2 ± 70.4	0.858	0.320	0.959
IL-17 (pg/mL)							
Initial	21 ± 20	21 ± 14	30 ± 50	32 ± 56			
Relative changes from initial (%)	8.6 ± 31.4	− 1.9 ± 25.4	9.1 ± 45.6	12.5 ± 45.7	0.475	0.324	0.070
G-CSF (pg/mL)							
Initial	709 ± 1234	638 ± 872	838 ± 1299	852 ± 1295			
Relative changes from initial (%)	5.2 ± 42.5	5.1 ± 34.9	9.1 ± 45.8	13.7 ± 44.4	0.749	0.392	0.488
GM-CSF (pg/mL)							
Initial	4 ± 3	3 ± 2	6 ± 7	6 ± 7			
Relative changes from initial (%)	− 46.8 ± 72.2	− 9.7 ± 101.0	3.4 ± 90.4	− 30.8 ± 57.3	0.749	0.392	0.488
INFγ (pg/mL)							
Initial	6 ± 4	5 ± 2	8 ± 8	8 ± 7			
Relative changes from initial (%)	9.2 ± 78.0	19.7 ± 80.4	26.2 ± 77.7	1.6 ± 77.4	0.722	0.919	0.207
MCP-1 (pg/mL)							
Initial	52 ± 29	51 ± 24	51 ± 25	54 ± 24			
Relative changes from initial (%)	− 0.7 ± 37.5	0.6 ± 29.7	9.4 ± 33.0	1.4 ± 40.3	0.628	0.379	0.477
MIP-1β (pg/mL)							
Initial	1496 ± 769	1506 ± 828	1445 ± 646	1403 ± 679			
Relative changes from initial (%)	1.5 ± 55.4	− 2.9 ± 60.6	10.1 ± 49.0	4.8 ± 53.6	0.383	0.768	0.743
TNFα (pg/mL)							
Initial	45 ± 49	45 ± 47	47 ± 49	48 ± 50			
Relative changes from initial (%)	1.6 ± 25.2	6.2 ± 15.8	6.6 ± 29.7	0.3 ± 23.1	0.953	0.911	0.203

Values represent the initial (pre-treatment) mean values and relative changes from initial values expressed as percentage ± SD. Relative changes from initial values were calculated from initial and final values as [(final value-initial value)/initial value] and analysed using a linear mixed model. *P* values in the first column correspond to the effect of taking OPO or HOSO, those of the penultimate column to the effect of the group (NC or HC), and the last column to the interaction of oil and group. Significance level was *P* < 0.05. *CRP* C reactive protein, *IL* interleukin, *G-CSF* granulocyte colony-stimulating factor, *GM-CSF* granulocyte–macrophage colony-stimulating factor, *IFNγ* interferon gamma, *MCP-1* monocyte chemoattractant protein-1, *MIP-1β* macrophage inflammatory protein 1 beta, *TNFα* tumour necrosis factor alpha

## Discussion

Most of the evidence on the potential health beneficial effects of olive minor components come from in vitro studies in different cell models or from preclinical animal studies [reviewed in 6]. However, clinical studies in humans on the cardiometabolic effects of these compounds are scarce, limited to the use of pure components such as squalene or triterpenic acids as drug therapy or dietary supplements [11, 12, 22]. More recently, some intervention studies assessed the effect of VOO enriched in triterpenes in healthy adults [13] or a blend of virgin and refined olive oils enriched with OA in prediabetic individuals [14]. But the effect of consuming OPO has only been addressed in postprandial studies, showing that OPO intake resulted in triglyceride-rich lipoproteins (TRL) with higher particle size than after the intake of ROO and facilitated TG clearance from TRL [23, 24]. Nevertheless, the effects on health of consuming OPO in the diet are still unknown. Here we report results from the first human intervention study with OPO, carried out in healthy individuals as well as in hypercholesterolemic subjects as a group at risk of suffering cardiovascular diseases.

Some of the most notable results observed were the decreased serum concentrations of LDL-C (*P* = 0.003), Apo B (*P* = 0.022) and LDL/HDL ratio (*P* = 0.027), with a trend to decrease TC (*P* = 0.064) (Table 3 and Fig. 2S). In a study carried out in hyperlipidemic patients consuming OA tablets, lower concentrations of TC, TG and LDL-C were observed after 4 weeks, although the statistical significance of this reduction was not established, nor the amount of OA administered to volunteers [11]. Indeed, pentacyclic triterpenes, secondary metabolites abundant in the unsaponifiable fraction of OPO, may have contributed to the reduction in plasma lipids observed in the present study, since they have been suggested to play a role in the treatment and management of CVD [25]. In the NUTRAOLEUM study, an optimized virgin olive oil (OVOO) enriched in phenolic compounds and a functional olive oil (FOO) enriched in both phenolic compounds and triterpenes were compared with VOO in healthy adults; none of the oils showed any effect on TC or LDL-C after daily consumption of 30 mL for 3 weeks, and only OVOO caused a significant increase in HDL-C [13]. Trials in several animal models have also reported reductions in serum TC, TG, LDL-C or VLDL-C and/or increased HDL-C concentrations following oral administration of different doses (15–100 mg/kg/d) of MA [reviewed in 26], OA (5–25 mg/kg/d) [25–29] or high (200 mg/kg/d) but not low (50 mg/kg/d) doses of ursolic acid (UA) [30]. However, results from these animal studies cannot be extrapolated to humans, since the amounts of triterpenic acids fed to experimental animals were much higher than those consumed by the volunteers in the present study, amounting to less than 10 mg/d.

Squalene is another characteristic component of olive oils with potential cardioprotective effects. Of the three trials developed in hypercholesterolemic patients, only one reported reduced TC and LDL-C, and increased HDL-C concentrations when participants were supplemented with 869 mg/d of squalene for 20 weeks [22]. These amounts of squalene are closer to pharmacological treatments and much higher than those derived from dietary consumption of oils naturally containing squalene, such as in the present study, where the daily intake of 45 g of OPO provided around 36 mg of squalene. On the other hand, animal trials have shown that squalene might have cardioprotective effects mostly due to its antioxidant properties, modulating atheroma plaque formation in mice, although its potential hypocholesterolemic effects varied depending on the animal model and the amount of squalene fed [31].

Aliphatic alcohols are present in OPO (978 mg/g) in much higher concentrations than in HOSO (32 mg/kg). A review by Hargrove et al. [32] suggests that consumption of 5–20 mg per day of mixed C24-C34 alcohols reduced LDL-C and increased HDL-C concentrations. Considering that OPO provided about 44 mg/d of aliphatic alcohols in the present study, these compounds might have contributed to the observed reduction of LDL-C concentrations. In contrast to aliphatic alcohols, tocopherols and total sterols are found in similar concentrations in the OPO and HOSO; thus, although they might have beneficial effects on cardiovascular health, they would not be responsible for the different responses observed after consuming OPO or HOSO.

Indeed, after HOSO intervention, there was an increase in TC, LDL-C, LDL/HDL and TC/HDL in both NC and HC volunteers, contrary to that observed with OPO (Table 3). This was in accordance with results from an animal experiment using high-fat diets supplemented with EVOO, HOSO and SO, showing that TC and LDL-C increased, and HDL-C decreased in comparison with control animals, suggesting no effect of the phenolic fraction nor the MUFA-rich content of EVOO and HOSO [33].

It is important to highlight the effects observed after OPO intake on coronary event predictors such as LDL/HDL ratio and Apo B concentrations. Apo B is considered a better predictor of cardiovascular risk than LDL-C [34]. As shown in Table 3, Apo B concentrations decreased following OPO consumption in healthy and at-risk participants. This is in line with the lower Apo B concentration in postprandial TRL after ingestion of OPO by healthy subjects compared to ROO [24].

Blood pressure showed no changes throughout the study, and SBP and DBP values remained within a normal range (Table 4). Several minor OPO components such as pentacyclic triterpenes (mainly OA), squalene or aliphatic alcohols (mainly octacosanol) have been associated with beneficial effects on blood pressure dysregulation [7, 35–37]. However, most of these assays were performed in vivo in hypertensive animals or in vitro using aorta rings isolated from hypertensive animals. In an intervention trial in patients diagnosed of metabolic syndrome, oral supplementation with 150 mg/d of UA for 12 weeks did not affect SBP or DBP [12], in line with the results obtained in the present study. On the other hand, it is considered that MUFA contribute to the prevention and maintenance of blood pressure in the general population [38]; therefore, no changes were expected in this regard, also considering that volunteers were normotensive subjects (Table 1).

Endothelial dysfunction is another key risk factor for atherosclerosis. None of the soluble molecules analysed as biomarkers of endothelial function showed significant changes, although circulating concentrations of eNOS increased after OPO and HOSO in both groups of volunteers (Table 5). Considering the role of eNOS on nitric oxide (NO) synthesis, which is a determining factor in vascular regulation due to its potent vasodilator action, the observed effect on eNOS, especially after OPO intake, might suggest a potential beneficial role of OPO on endothelial function. Previous animal studies have reported that OPO or its triterpene fraction (mainly OA) improve endothelial function in both aorta and mesenteric arteries by increasing eNOS expression [7]. Triterpenic acids (OA, MA and UA) have been reported to suppress the expression of E-selectin, ICAM-1 and VCAM-1 in different cultured human endothelial cells [39–41], although none of these parameters showed variations after the intake of OPO and HOSO (Table 5).

Flow mediated dilatation (FMD) is a non-invasive measurement of vasodilation and it is considered an index of NO bioavailability [42]. Since measurement of FMD requires operator experience and skill, a similar automated technique for measuring endothelial function assessing the hyperemic response, known as peripheral arterial tonometry (PAT), was used in this trial [43]. Although peripheral endothelial function has been often evaluated (with the FMD technique) in studies with olive oil, the potential effect of OPO on this marker is so far unknown. A meta-analysis including 3106 subjects showed an increase in FMD in participants who received an olive oil-enriched diet [44], confirming the beneficial effects of MUFA and a potential contribution of olive oil phenolic compounds on FMD. However, in the present study PAT was not significantly changed (Table 5), and only a trend to increase PAT was observed. This is in line with results from a recent study in 53 subjects at moderate risk of CVD, where an increase in FMD was observed when consuming dairy products fortified with MUFA for 12 weeks [45].

Another underlying mechanism of endothelial dysfunction is inflammation. As shown in Table 6, no significant changes occurred after dietary intervention with both oils (OPO and HOSO). It should be remembered that, despite the high cholesterol concentrations in the at-risk group, the participants were otherwise healthy, without an established inflammatory state. Therefore, no major changes in inflammatory biomarkers were expected. Previous assays have shown that triterpenes characteristic of OPO (OA and MA) modulate endothelial cell inflammation and suppress the production of inflammatory mediators such as TNFα and nuclear transcription factor kappa B (NF-κB) in in vivo and in vitro assays [7, 8, 34, 39]. These trials were carried out in lipopolysaccharide challenged endothelial cells [39] or in animal models of different pathologies such as hypertension [7] or obesity [8], involving inflammatory processes.

Overall, consumption of OPO during four weeks resulted in an improved blood lipid profile, decreasing LDL-C, Apo B and LDL/HDL both in healthy and at-risk volunteers, in contrast to the opposite effect observed with HOSO, with no significant changes in other CVD risk factors. It is important to highlight that this study intended to reproduce normal consumption patterns in which OPO was used as culinary oil, without further supplementing or enriching this oil with bioactive compounds, either phenolics, triterpenes, squalene, etc. This resulted in relatively low intakes of the minor components of olive unsaponifiable fraction, which might have compromised observing more clear responses on the different biomarkers studied.

This study has several strengths and limitations. The study was well-powered, and its randomized, controlled, crossover design can be considered as another strength. The dietary intervention had a realistic approach, since participants consumed a moderate amount of oil in accordance to Spanish recommendations. OPO was a non-supplemented oil as consumers can find in supermarkets. As for limitations, SO intake in the run-in and wash-out periods resulted in a different lipid profile (MUFA, PUFA, and SFA) intake in comparison with the test and control oils. However, the crossover design ensured that all subjects started with the same dietary conditions at the beginning of each intervention. NO could not be determined in blood samples. Volunteers were instructed to maintain their lifestyle unchanged; however, physical activity was not surveyed during the study. To assess endothelial function, PAT, a non-invasive technique similar to FMD, was used to measure the hyperemic response. This had certain limitations considering that the data generated by endoPAT have more variability in their results compared to the FMD technique [43].

In conclusion, OPO could have hypolipidemic actions in healthy consumers and in subjects with high blood cholesterol, contributing to cardiovascular disease prevention. This is the first chronic clinical intervention carried out with OPO and its results corroborate previous findings with some of the characteristic minor components of this oil. Therefore, this study contributes to consider OPO as a relevant source of fat in the diet.

## Supplementary Information

Below is the link to the electronic supplementary material.

Supplementary file1 (DOCX 164 KB)

## Data Availability

Data described in the manuscript may be made available upon request pending application and approval.
