# Supplementary material for: Olive pomace oil can improve blood lipid profile: a randomized, blind, crossover, controlled clinical trial in healthy and at-risk volunteers
Source: Eur J Nutr. 2022 Sep 24;62(2):589–603. doi: 10.1007/s00394-022-03001-y (PMC9941261; doi:10.1007/s00394-022-03001-y)
Supplement: Supplementary file 1 — Supplementary file1 (DOCX 164 KB) [file 394_2022_3001_MOESM1_ESM.docx]

**Supplementary Information**

**Olive pomace oil can improve blood lipid profile: a randomized, blind, crossover, controlled clinical trial in healthy and at-risk volunteers.**

***European Journal of Nutrition***

Susana González-Rámila, Beatriz Sarriá, Miguel A. Seguido, Joaquín García-Cordero, Raquel Mateos, Laura Bravo*

Institute of Food Science, Technology and Nutrition (ICTAN-CSIC). Spanish National Research Council (CSIC), José Antonio Nováis 10, 28040 Madrid. Spain.

**Supplementary Table 1S.** Chemical composition of olive pomace oil (OPO), high oleic sunflower oil (HOSO) and sunflower oil (SO).

|  | **OPO** | **HOSO** | **SO** |
| --- | --- | --- | --- |
| **Fatty acids (%)** | | | |
| C 14:0 (Myristic) | 0.02 | 0.0 | 0.1 |
| C 16:0 (Palmitic) | 11.63 | 4.2 | 6.4 |
| C 16:1 (Palmitoleic) | 0.98 | 0.1 | 0.2 |
| C 17:0 (Margaric) | 0.07 | 0.0 | 0.1 |
| C 17:1 (Margaroleic) | 0.11 | 0.1 | 0.1 |
| C 18:0 (Stearic) | 2.83 | 3.3 | 3.6 |
| C 18:1 (Oleic) | 71.01 | 76.5 | 29.6 |
| C 18:2 (Linoleic) | 11.5 | 13.9 | 58.6 |
| C 20:0 (Arabic) | 0.49 | 0.3 | 0.3 |
| C 18:3 (Linolenic) | 0.76 | 0.1 | 0.1 |
| C 20:1 (Eicosenoic) | 0.34 | 0.2 | 0.2 |
| C 22:0 (Behenic) | 0.19 | 1.0 | 0.7 |
| C 24:0 (Lignoceric) | 0.07 | 0.3 | 0.2 |
| *Trans* Oleic (t-C 18:1) | 0.32 | 0.0 | 0.0 |
| *Trans* Linoleic + *Trans* Linolenic (*t*-C18:2 + *t*-C18:3) | 0.1 | 0.2 | 0.4 |
| **Triglycerides (%)** | | | |
| C 46 | <0.05 | <0.05 | <0.05 |
| C 48 | 0.14 | <0.05 | <0.05 |
| C 50 | 4.54 | 1.34 | 0.66 |
| C 52 | 32.38 | 19.17 | 12.93 |
| C 54 | 61.43 | 77.73 | 83.96 |
| C 56 | 1.27 | 0.92 | 1.16 |
| C 58 | 0.24 | 0.84 | 1.29 |
| C 60 | <0.05 | <0.05 | <0.05 |
| C 62 | <0.05 | <0.05 | <0.05 |
| Squalene (ppm) | 799 | 87 | 117 |
| **Tocopherols (mg/kg)** | | | |
| α-Tocopherol (vit E) | 357 | 420 | 518 |
| β-Tocopherol | <2 | 40 | 44 |
| γ-Tocopherol | 32 | 72 | <2 |
| δ-Tocopherol | <2 | 51 | <2 |
| **Sterols (%)** | | | |
| Cholesterol | 0.1 | 0.1 | 0.1 |
| Brassicasterol | <0.1 | <0.1 | <0.1 |
| 24-Methylcholesterol | 0.2 | 0.2 | 0.3 |
| Campesterol | 3.2 | 8.7 | 8.7 |
| Campestanol | 0.1 | 0.1 | 0.1 |
| Stigmasterol | 1.2 | 8.4 | 7.8 |
| Δ7-Campesterol | <0.1 | 2.9 | 2.7 |
| Δ5,23-Estigmastadienol | 0.8 | 0.2 | 0.1 |
| Clerosterol | 1.5 | 0.8 | 0.7 |
| β-Sitosterol | 86.1 | 54.1 | 54.6 |
| Sitostanol | 1.7 | 0.6 | 0.5 |
| Δ5-Avenasterol | 1.6 | 3.6 | 3.4 |
| Δ5,24-Stigmastadienol | 2 | 1.1 | 1 |
| Δ7-Stigmastenol | 0.4 | 13.5 | 14.5 |
| Δ7-Avenasterol | 1 | 5.7 | 5.5 |
| β-Sitosterol apparent | 93.8 | 60.5 | 60.3 |
| Total Sterols (ppm) | 2839 | 3040 | 3315 |
| **Triterpenic alcohols (mg/kg)** | | | |
| Erythrodiol + Uvaol | 886.6 | <1.0 | <1.0 |
| **Phenols** **(mg/kg)** | | | |
| Total phenols | < 1.0 | < 1.0 | < 1.0 |
| **Triterpenic acids (mg/kg)** | | | |
| Oleanolic acid | 187.3 | <2.0 | <2.0 |
| Ursolic acid | 6.9 | <2.0 | <2.0 |
| Maslinic acid | 2.3 | <2.0 | <2.0 |
| **Aliphatic alcohols (mg/kg)** | | | |
| C22+C24+C26+C28 | 978 | 32 | 26 |

**Chemical characterization of the study oils**

The oils used in the study were analyzed according to the following standardized methods: ISO 12228-2:2014 method for the determination of sterols; Regulation (EEC) No. 2568/91 Annex V for determining triterpenic alcohols; Regulation (EEC) No. 2568/91 Annex XIX for determining aliphatic alcohols; Regulation (EEC) No. 2568/91 Annex X for determining fatty acid composition; and ISO 9936:2016 for determining tocopherols and tocotrienols. Triterpenic acids were analyzed following the method of Pérez-Camino & Cert (1999) (1), and squalene was determined by gas chromatography (Giacometti, 2001) (2). Phenols were analyzed by high-performance liquid chromatography with on-line diode array detection (HPLC-DAD) according to the procedure by Mateos et al. (2001) (3).

1. Pérez-Camino MC, Cert A (1999) Quantitative determination of hydroxy pentacyclic triterpene acids in vegetable oils. J Agric Food Chem 47:1558-1562.
2. Giacometti J (2001) Determination of aliphatic alcohols, squalene, alpha-tocopherol and sterols in olive oils: direct method involving gas chromatography of the unsaponifiable fraction following silylation. Analyst 126:472-475.
3. Mateos R, Espartero JL, Trujillo M, Ríos JJ, León-Camacho M, Alcudia F, Cert A (2001) Determination of phenols, flavones, and lignans in virgin olive oils by solid-phase extraction and high-performance liquid chromatography with diode array ultraviolet detection. J Agric Food Chem 49:2185-2192.

Olive Pomace Oil (**OPO**)

High Oleic Sunflower Oil (**HOSO**)

Sunflower Oil (**SO**)

*Run-in*

*Wash-out*

*Intervention 1*

**OPO**

**HOSO**

**SO**

*Intervention 2*

Week 1

Week 3

Week 7

Week 10

Week 14

**Supplementary Figure 1S** Timeline of the nutritional intervention with olive pomace oil (OPO), high-oleic acid sunflower oil (HOSO). Normal sunflower oil (SO) was used during run-in and wash-out.

**
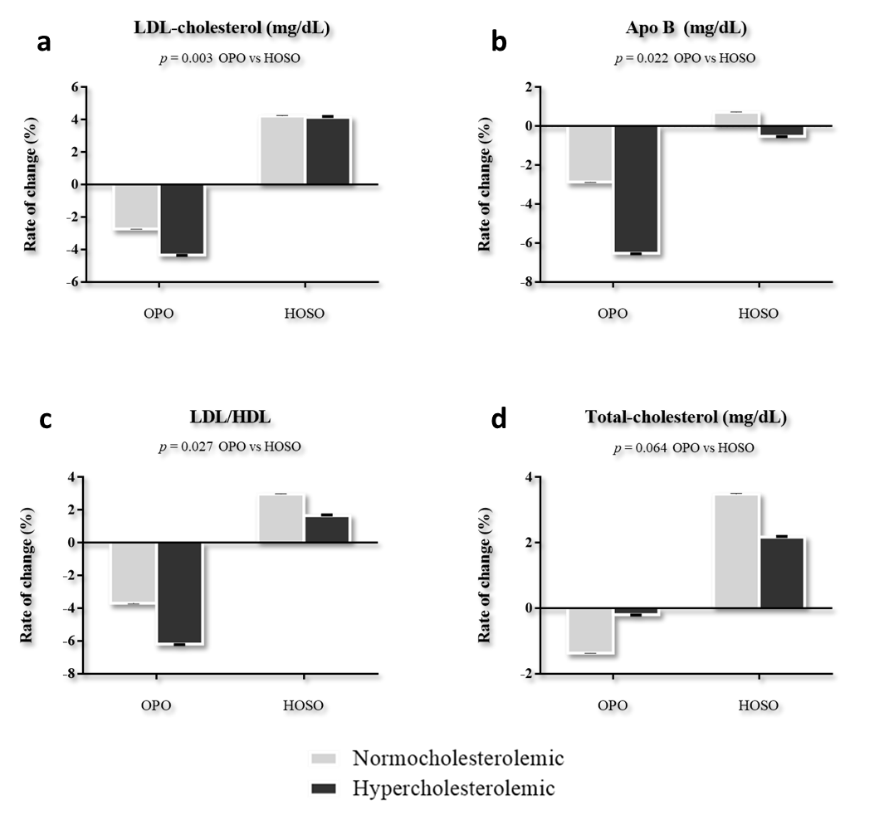
**

**Supplementary Figure 2S** Values represent mean ± SEM of (a) LDL-cholesterol, (b) Apolipoprotein B, (c) LDL/HDL and (d) Total-cholesterol. The rate of change was calculated from initial and final values as [(final value-initial value)/initial value]. Data were analyzed using a linear mixed model. *p* values in the first column correspond to the effect of taking the oil (olive pomace oil -OPO- or high oleic sunflower oil -HOSO-).
